# Spectrum of intra-thoracic lesion detected by computed tomography guided fine needle aspiration biopsy

**DOI:** 10.1186/1755-7682-6-4

**Published:** 2013-02-12

**Authors:** Hanna Naqvi, Muhammad Muzzammil Edhi, Hafiz Muhammad Aslam, Naveen Faridi

**Affiliations:** 1 Liaquat National Medical College, Liaquat National Hospital, Karachi, Pakistan; 2 Dow Medical College (DUHS), Flat #14, 3rd floor, Rafiq Mansion, Cambell road, Off Arambagh, Karachi, Pakistan

**Keywords:** Computed tomography, Intrathoracic lesion, Cytology

## Abstract

**Background:**

Fine needle aspiration biopsy (FNAB) is a rapid, sensitive and inexpensive procedure for diagnosing benign and malignant palpable lesions. For lesions that are not palpable or deep seated, FNAB can be performed under the guidance of radiological imaging. Our basic objective was to evaluate the spectrum of intrathoracic lesions by using Computed Tomography guided fine needle aspiration biopsy and evaluate its diagnostic yield.

**Methodology:**

It was a retrospective study carried out in the Department of Histopathology, Liaquat National Hospital and Medical College, during the months of August 2011 and August 2012. All patients with pulmonary, mediastinal or paravertebral mass who underwent CT guided intrathoracic biopsy were included in this study. Fine needle aspiration biopsies were performed in the Radiology Department and specimen retrieved was sent in 10% buffered Formalin to the Histopathology Department. All the data was entered and analyzed through SPSS 19.0.

**Results:**

A total of 130 cases were evaluated, out of which 108 (83.1%) were pulmonary, 16 (12.3%) were mediastinal and 6 (4.6%) were paravertebral. Conclusive biopsies were possible in 113 cases, while 17 biopsies were inconclusive. In those that showed a conclusive diagnosis, 83.1% were neoplastic and 16.9% were non neoplastic. Of the neoplastic cases, 27 (20.8%) were adenocarcinomas, followed by squamous cell carcinomas (15.4%) and large cell carcinoma, not otherwise specified, (12.3%).

**Conclusion:**

CT guided fine needle aspiration biopsy is a reliable tool for examination of intrathoracic lesions, with a high rate of conclusive diagnosis.

## Introduction

Fine needle aspiration biopsy (FNAB) is a rapid, sensitive and inexpensive procedure for diagnosing benign and malignant palpable lesions. For lesions that are not palpable or deep seated, FNAB can be performed under the guidance of radiological imaging [1,2]. In previous days, open biopsy of intrathoracic mass was done to reach a diagnosis but as the medicine is evolving the newer modalities are their to image the body and guide the Radiologist, open biopsy has become almost obsolete now [3]. Ultrasonography was commonly used as image guiding tool towards the target lesion. However, Haaga and Alfidi reported computed tomography (CT)-guided biopsy in 1976, and numerous reports since that time have shown FNAB procedures to be both effective and accurate. The diagnostic accuracy has been reported as greater than 80% for benign disease and greater than 90% for malignant disease [4]. CT guidance permits biopsy of nearly all lesions that are visible on CT scans, regardless of size or position. Needle placement in small pulmonary lesions or deep mediastinal nodes can be accurately determined with CT, and vascular and cardiac structures are well demonstrated and safely avoided [5]. Fine needle aspiration biopsy provides aspirates for cytological analysis and also tissue core for histopathological evaluation. The role of FNAB is predominantly to differentiate between benign and malignant lesion while there are possibilities of false positive or false negative result [6].

The most recent study done by Gupta et al. in 2012 showed a diagnostic accuracy of 91% for the biopsy of intrathoracic lesions using ct guidance. The diagnostic sensitivity of transthoracic FNAB for malignant lesions ranges from 76% to 97% [7-9].

The main aim of our study was to evaluate the pathological spectrum of intrathoracic lesions by using computed tomography guided fine needle aspiration biopsies and to determine its diagnostic accuracy as no relevant study has been done in our region which would help to assess the significance of this minimally invasive procedure.

## Methodology

It was a retrospective study carried out in the dept of Histopathology, LNH and Medical College. Duration of the study was of 1 year extending from August 2011 to August 2012. Total 130 patients were evaluated. Written consent was taken from all the patients. Inclusion criteria were:

1. All CT guided biopsies of intrathoracic lesions sent to the dept of Histopathology.

2. Specimen received in formalin.

3. Biopsies of patients between ages 15–70 years.

Exclusion criteria were:

1. Biopsies that were not sent in formalin.

2. Biopsies performed under radiological modalities other than Ct guidance.

Study was approved from the Institutional Review Board of Liaquat National Medical College. The coagulation parameters and platelet counts were obtained in all patients to exclude any bleeding diathesis. Results of contrast material–enhanced CT was available for evaluation prior to biopsy in all cases. Biopsies were performed by 2 expert radiologist of Liaquat National Hospital, by placing participant in supine, prone or in lateral decibutus position depending on the location of lesion and safe approach for the placement of needle. All biopsies were performed by using a computed tomography to localize the location and size of target lesion. Patients initially underwent imaging in the supine position with a section thickness of 5, 7, or 10 mm. Patients also underwent contrast-enhanced CT after intravenous administration of ioversol to define the major vessels in the vicinity of the lesions. Once the access route and needle entry site were chosen, the skin was prepared in a sterile fashion, and 1% lidocaine hydrochloride was administered with a 25- gauge hypodermic needle to anesthetize the skin, subcutaneous tissue, and periosteum of the anterior sternal cortex. Fine needle aspiration was performed by using 22 gauge spinal needles. Cores were obtained by using an 18 gauge Truecut biopsy needle. Needle was mostly between 11–22 gauges. The aspiration/biopsy needle was advanced in a coaxial fashion through the needle guide into the lesion during suspended respiration. Transpulmonary route has been used for the lesion in lung and transsternal route for mediastinal mass.

After the needle tip was confirmed to be in the desired location by taking the limited CT cuts, aspiration/biopsy was performed. Each fine needle aspirate was immediately smeared onto glass slides; air dried, fixed with 95% ethyl alcohol and immediately sent for cytological analysis. The biopsy specimen were preserved in formalin and sent for histopathology. One to four samples were required in each patient. Each participant was observed for 1–3 hours after the procedure to ensure the hemodynamic stability and to monitor their respiratory status. Procedure was performed on the outpatient basis unless the participant was already hospitalized. At the completion of the procedure, all patients underwent expiratory chest radiography to detect complications like pneumothorax and were monitored for 3 to 4 hours in the recovery area. The specimen was taken in 10% buffered formalin and sent to the hispathological department.Here the sample was placed in respective cassettes and processes in concentration of alcohol, embedded in paraffin and stained over glass slide using Hematoxylin Eosin dye. These slides were reviewed by 2 expert pathologist in which one have the experience of 20 years and other have 4 years of experience, both were also the part of study.

All the data was entered and analyzed through SPSS 19. Mean and standard deviation was calculated for continuous data. Frequency and percentage were calculated for the categorical data.

## Results

CT guided transthoracic fine needle aspiration biopsy was performed in 130 patients. Out of which 83(63.8%) were males and 47(36.2%) were females. Mean age was 54.52 ± 16.15 years and majority of the cases seen in 6^th^ and 7^th^ decade (Table 1).

**Table 1:** Table represents the demographic area, age groups and site of lesions

**S.no**	**Variables**	**Frequency**	**Percentage**
1	Gender		
A	Male	83	63.8
B	female	47	36.2
2	Age groups		
A	1–10 years	0	0
B	11–20 years	2	1.55
C	21–30 years	16	12.25
D	31–40 years	11	8.5
E	41–50 years	25	19.2
F	51–60 years	27	20.8
G	61–70 years	33	25.4
H	70–80 years	16	12.3
3	Region of lesion		
A	Pulmonary lesion	108	83.1
B	Mediastinal lesion	16	12.3
C	Vertebral lesion	6	4.6

Majority of cases was present in the age groups of 60–70 years 33(25.4%), 50–60 years 27(20.8%) and 40–50 years 25(19.2%) (Table 1).

FNAB specimen was considered adequate for specific diagnosis by the histopathological staff in 86.92% of lesion. Diagnostic yield (113/130 = 86.92%) was 86.92%.

Out of total 130 cases, 108(83.1%) had lesion in lungs while 16(12.3%) had lesion in mediastinum. 6 (4.6%) cases were of vertebral region (Table 1).

All cases were divided into two categories malignant and benign. In malignant, 27(20.8%) cases were of adenocarcinoma, while 20(15.4%) cases had squamous cell carcinoma, 16(12.3%) were had large cell carcinoma, neoplastic lesion was detected in 15(11.5%) cases. In benign cases, necrosis and inflammation was found in 13(10%) cases and chronic granulumatous inflammation was recorded in 9(6.9%) cases (Table 2).

**Table 2:** Table represents the malignant, benign and non-neoplastic lesion

**S.no**	**Variables**	**Frequency**	**Percentage**
1	Malignant		
A	Adenocarcinoma	27	20.8
B	Squamous cell carcinoma	20	15.4
C	Large cell carcinoma	16	12.3
D	Neoplastic lesion	15	11.5
E	Small cell carcinoma	4	3.1
F	Thyomyoma	2	1.5
G	Metastatic carcinoma	2	1.5
H	High grade carcinoma	2	1.5
I	Pre lymphoblastic lymphoma	1	0.8
J	Plasma cell neoplasm	1	0.8
K	Repeat	17	13.1
2	BENIGN		
A	Necrosis and Inflammation	13	10.0
B	Chronic granulomatous inflammation	9	6.9
C	Benign thyroid tissue	1	0.8

In pulmonary biopsies, 26(24.1%) had adenocarcinoma, 20(18.5%) had squamous cell carcinoma, 14(13%) had large cell carcinoma and necrosis and inflammation were detected in 12(11.1%) (Table 3).

**Table 3:** Table represents the pleural, mediastinal and vertebral diagnosis

**S.no**	**Variables**	**Frequency**	**Percentage**
1	Pleural lesion diagnosis	108	100
	Adenocarcinoma	26	24.1
	Squamous cell carcinoma	20	18.5
	Large cell carcinoma	14	13.0
	Necrosis and inflammation	12	11.1
	Neoplastic lesion	10	9.3
	Chronic granulamotous	8	7.4
	Small cell carcinoma	3	2.8
	Thymoma	1	0.9
	Metastatic carcinoma	1	0.9
	Repeat	13	12
2	Mediastinal lesion diagnosis	16	100
	Neoplastic lesion	5	31.3
	Adenocarcinoma	1	6.3
	High grade carcinoma	2	12.5
	Thyomoma	1	6.3
	Large cell carcinoma	1	6.3
	Small cell carcinoma	1	6.3
	Benign thyroid tissue	1	6.3
	Pre t lymphoblastic cell lymphoma	1	6.3
	repeat	3	18.3
3	Vertebral lesion diagnosis	6	100
	Necrosis and inflammation	1	16.7
	Metastatic carcinoma	1	16.7
	Chronic granulumatous inflammation	1	16.7
	Large cell carcinoma	1	16.7
	Plasma cell neoplasm	1	16.7
	repeat	1	16.7

In mediastinal biopsies, neoplastic lesion were recorded in 5(31.3%), while 2(12.5%) had high grade carcinoma (Table 3).

## Discussion

FNAB is now universally accepted as the procedure of diagnosis of patients when both clinical data and imaging is combined. It is used to evaluate number of pathologies in various organs like thyroid, breast, lymph node and prostate [2,10]. Procedural requirement of FNAB is the presence of alert histopathologist to evaluate the diagnosis [11]. The main goal of early diagnosis by CT guided needle biopsy is to separate the benign from malignant lesion and decrease the load of mortality and morbidity. Prior to this technique world depends upon other procedures in which there are more risk of complication and anatomically critical sites poses great difficulties. Compared to other procedures CT guided biopsies are of low cost because they reduced the time period from presence to diagnosis. It also decreases the number of surgical procedures and reduces the time for hospital stay [6]. In addition to high specificity and sensitivity, FNAB has numerous advantage, such as lower cost, outpatient technique, minimally invasive approach and possibility of obtaining material for special histochemical of immuno histochemical staining, electron microscopy, flow cytometry and culture, to use these adjuvant test it is essential that aspirational material be adequate in quality and quantity to make accurate diagnosis [12].

In our study mostly cases were evaluated in 6^th^ and 7^th^ decade which were in contrast to previous study [13]. Evaluation of majority of cases occur in old age certify the common fact that mostly chronic infection and malignancies occur in old age. This is due to accumulation of mutation, wear and tear, antagonistic pleiotrophy and susceptibility of old tissue to more carcinogen and decline in proliferation of cells, these all facts make a favorable environment for a chronic infection and malignancies [2].

It was detected in our study that mostly cases were in the age groups of 61–70 years which was same as shown in previous study [13]. The main objective of the aspirational and histological diagnosis were to differentiate between malignant lesion and benign. Many reason to consider this ability is to provide a proper diagnosis pre operatively in order to start therapeutic planning specially in patients with significant co-morbidities and where the significant risk were high. Fascinatingly in our study malignant lesion was found in much more quantity than the benign. This result was comparable with other international and local studies [6,13,14].

On of the major disadvantage of FNAB is unsatisfactory aspiration. There were many factors contributing this like non satisfactory training of radiologist and pathologist and it was seen that the main factor contributing in obtaining samples in our study was different tumor location, site of aspiration, operator experience and interpretative skills of pathologist, Among 130 cases 17 were inadequate and this was much high than past study, but was also lesser than a study conducted in Norway [15].

In our study it was detected that the most common tumor of lung is adenocarcinonma followed by squamous cell carcinoma and small cell carcinoma as indicated in past studies [16,17]. Necrosis and inflammation of lung was also found in our study and this finding was consistent with other study [1], but frequency and percentage of lesion was much less than a study conducted in Herzegovina [1].

Thymomas is one of the common cause of thymic mass and it is one of the most common primary mediastinal neoplasm, also detected in our study, while other mediastinal mass which is recorded in FNAB are thymic carcinoma which is large infiltrating mass with area of cystic damage and necrosis and it is of squamous cell type**,** It was also indicated that Pre-T cell lymphoblastic lymphoma was also present as mediastinal mass. These findings were comparable with other studies [18,19].

Unlikely in our study, mostly mediastinal masses were of high grade or either they can’t be differentiated into benign or malignant. Diagnoses of these masses were quiet challenging because of the difficulty in obtaining good biopsies and due to obtaining of small and crush biopsies.

In our study we use transternal approach for the masses in mediastinum. It was successfully done in all patients as compare to past study [14]. It is the experience and concluded in our study that trans-sternal approach is best for anterior mediastnal masses and for anterior and medial aspect of lungs. In this technique, access to the middle and post mediastinal mass behind great vessels in the pre-tracheal compartment or in the aortopulmonary window can be achieved.

In our study, diagnoses of biopsies reveals the presence of squamous cell carcinoma, adenocarcinoma, large cell carcinoma, small cell carcinoma and chronic granulumatous inflammation same as indicated in past studies but with different frequencies and percentages [2,4].

Interestingly our study also reveals the presence of plasma cell neoplasm, pre-T cell lymphoblastic lymphoma, and necrosis and inflammation of mediastinal and pulmonary mass which were not indicated in past studies and these finding make our study unique in this way.

### Limitations

Several limitations of the present study should be considered. First and most important is that our sample size was small. 2^nd^, study was conducted in one tertiary care hospital of Karachi and data cannot predict the overall situation in the country.

### Recommendation

• It is recommended that healthy collaboration between radiologist and pathologist while performing image guided FNAB in deep region is necessary.

• It is also recommended that lesion of patients which is seen in chest radiograph should be discussed with respiratory physician and radiologist; operator should take steps and try to audit their own practice and achieve the lowest complication rates.

• PT, APTT, platelet count, and pulmonary function tests are desirable before needle biopsy. In patients with risk factors for bleeding, PT, APTT and platelet count should be done.

• “High risk” patients should not have a biopsy performed as a day case procedure.

• Patients should be warned of delayed complications and given verbal and written instructions to return if symptomatic.

• Further studies should be conducted on a larger scale, with a more diverse set of institutes in order to minimize bias and for better generalization.

## Conclusion

It has been shown in our study that CT guided biopsies of mediastinal and pulmonary masses are relatively safe, simple and well tolerated. This technique reduces the patient tenure of hospitalization and lowering the cost of health care. There are very few complications and multiple specimens can be obtained without increasing morbidity.

## Abbreviation

FNAB: Fine needle aspiration biopsy.

## Competing interest

All authors declared that they have no competing interest.

## Authors’ contribution

HN, MME and HMA did the work of paper writing, synopsis formation, data analyzing and data collection. NF: did critical review and guide as supervisor. All authors read and approved the final manuscript.
